# Graph theory-based analysis of functional connectivity changes in brain networks underlying cognitive fatigue: An EEG study

**DOI:** 10.1371/journal.pone.0329212

**Published:** 2025-08-04

**Authors:** Yabing Lou, Rui Pi, Ruifeng Sun, Jilin Wu, Wei Wang, Ziman Zhu, Tengteng Dai, Weijun Gong

**Affiliations:** 1 Chinese Medicine Department, Beijing Rehabilitation Hospital, Capital Medical University, Beijing, China; 2 Beijing Rehabilitation Hospital, Beijing Rehabilitation Medicine Academy, Capital Medical University, Beijing, China; 3 Department of Rehabilitation Radiology, Beijing Rehabilitation Hospital, Capital Medical University, Beijing, China; 4 The Second Clinical Medical College of Yunnan University of Chinese Medicine, Yunnan, China; 5 Department of Neurological Rehabilitation, Beijing Rehabilitation Hospital, Capital Medical University, Beijing, China; Hangzhou Normal University, CHINA

## Abstract

**Objective:**

This investigation was designed to analyze alterations in functional connectivity across brain networks associated with cognitive fatigue through electroencephalogram (EEG) data analysis. Through the application of both global and local graph-theoretical metrics to characterize the topology of brain networks, this study establishes a conceptual framework supporting enhanced detection of cognitive fatigue manifestations while facilitating examination of its neurophysiological substrates.

**Methods:**

The study cohort comprised neurologically intact individuals aged 20–35 years, recruited from Beijing Rehabilitation Hospital, Capital Medical University between February 6 and September 30, 2024 for participation in a cognitive fatigue induction task. Following acquisition of written informed consent, data before and after the task were obtained, including both subjective fatigue assessments using the Visual analog scale for fatigue (VAS-F) scores and EEG data. The preprocessed EEG signals were segmented into three frequency bands: θ (4–8 Hz),α (8–13 Hz), and β (13–30 Hz). To determine the frequency band exhibiting maximal sensitivity to cognitive fatigue, cross-band comparative power spectral density (PSD) was implemented. The selected frequency band subsequently served as the basis for weighted Phase Lag Index (wPLI) computation, yielding a functional connectivity matrix derived from wPLI measurements. Network topology was evaluated through application of five global graph theory metrics (global efficiency [Eg], local efficiency [Eloc], clustering coefficient [Cp], shortest path length [Lp], and small-world property [Sigma]) complemented by two local graph theory metrics (nodal efficiency [NE] and degree centrality [DC]). This analytical framework enabled systematic comparison of connectivity patterns and topological characteristics between before and after cognitive fatigue states.

**Results:**

Statistical analysis revealed significant post-fatigue elevations in global average PSD across all examined frequency bands: α (*p* < 0.001), θ (*p* < 0.001), and β (*p* = 0.004). The α band demonstrated the most pronounced effect size (Cohen’s d = 4.23, r = 0.90). Topological analysis of α-band wPLI networks showed enhanced Eg (*p* = 0.005), Eloc (*p* < 0.001), and Cp (*p* < 0.001), whereas Lp displayed significant reduction (*p* = 0.005). Regional analysis revealed preferential enhancement of NE, particularly in central and anterior cortical regions.

**Conclusion:**

The experimental data indicated that α-band activity exhibited the highest sensitivity to cognitive fatigue induced by the sustained Stroop task, establishing a framework for accurate identification of fatigue states. Cognitive fatigue compensatory mechanisms manifested as concurrent improvements in both local and global neural information processing efficiency. Although such adaptive reorganization may compromise overall network efficiency, these findings implied an inherent balance between adaptive network reconfiguration and system efficiency. These results elucidated novel neurophysiological mechanisms underlying cognitive fatigue, substantially advancing our understanding of brain network dynamics during prolonged cognitive demand.

## Introduction

Cognitive fatigue constitutes a neurophysiological condition induced by sustained engagement in cognitive tasks [1], manifesting as attentional deficits, reduced vigilance, and impaired cognitive processing efficiency [2,3]. This phenomenon transcends cognitive domains, exerting detrimental effects on physical functional capacity [4]. Research shows that individuals with cognitive impairments are particularly sensitive to cognitive fatigue, characterized by pronounced performance decrements and elevated subjective fatigue [5–7]. These fatigue-induced functional limitations adversely affect both therapeutic progress and patient-reported quality of life [8–11]. Consequently, elucidation of the neural basis of cognitive fatigue holds significant translational value for both healthy individuals and clinical populations.

Electroencephalogram (EEG) measurements have gained widespread recognition as a reliable physiological biomarker for cognitive fatigue assessment due to their capacity for direct measures of brain activity [12]. Existing literature documented consistent associations between cognitive fatigue and elevated θ-band activity coupled with attenuated α-band and β-band activity in EEG signals [13,14]. However, contradictory reports suggested potential α-band activity augmentation under fatigued conditions [15,16]. Despite these findings, research examining power spectral density (PSD) changes across various frequency bands induced by a single cognitive task remains insufficient. Additional studies are required to identify which frequency band changes are most sensitive for detecting fatigue.

In addition to frequency band analysis of neural activity, investigations into functional brain networks provide new insights into the understanding of cognitive fatigue. Functional brain networks comprise functionally integrated brain regions that work collectively to mediate specific cognitive processes through coordinated interactions [17]. These characteristics of functional connectivity in the brain constitute the neural basis for cognition and behavior [18,19]. Therefore, functional connectivity analysis represents an essential methodological approach for investigating alterations in brain network structure to advance our understanding of cognitive fatigue.

The weighted Phase Lag Index (wPLI) is an indicator used to quantify phase coupling between signals, particularly well-suited for handling non-stationary signals and noise interference, as it effectively suppresses random phase delays. Moreover, wPLI can diminish the impact of spurious phase coupling, thereby providing more reliable analytical results [20,21]. Previous studies have shown a significant association between brain network features constructed using wPLI and cognitive function [22,23]. Based on this, we hypothesize that wPLI is also suitable for revealing the changes in functional connectivity of specific brain regions induced by high-load tasks during cognitive fatigue. By utilizing functional connectivity metrics based on wPLI and incorporating graph theory analysis methods, researchers can gain deeper insights into the collaborative patterns among brain regions. This approach not only enhances our understanding of functional brain networks but also provides valuable tools and theoretical foundations for future research directions.

While substantial research efforts have been devoted to examining cognitive fatigue induced by prolonged monotonous tasks, particularly within simulated driving scenarios [24,25], relatively few investigations have addressed fatigue manifestations following continuous high-intensity cognitive exertion. The existing literature predominantly concentrates on the correlation between event-related potential (ERP) signals and cognitive fatigue [26,27], frequently overlooking systematic evaluation of functional connectivity patterns in brain networks. This study examines PSD variations in EEG signals across multiple bands before and after cognitive fatigue induced by a sustained Stroop task, with the primary goal of determining optimal biomarkers for cognitive fatigue detection. Functional connectivity networks will be constructed through wPLI computation, followed by quantitative analyses using graph-theoretical metrics. These analytical approaches seek to establish a theoretical foundation for the precise identification of cognitive fatigue states while elucidating the neural mechanisms underlying fatigue-related modifications in brain network topology and functional connectivity.

## Materials and methods

### Participants

All participants were recruited from Beijing Rehabilitation Hospital, Capital Medical University, with strict adherence to predetermined inclusion parameters: age between 20 and 35 years, right-handedness, absence of color vision deficiency, either uncorrected or corrected visual acuity within normal limits, and no documented neurological conditions or psychiatric disorders. To standardize baseline conditions, all participants were required to satisfy three inclusion criteria: (1) nocturnal sleep initiation prior to 23:00 on the pre-experimental night with minimum sleep duration maintained at ≥7 hours; (2) sustained relaxation for a 2-hour period preceding experimental procedures, including refraining from strenuous physical or mental activities; and (3) complete abstention from caffeine-containing consumables during the 2-hour pre-experimental interval. Participant enrollment occurred between February 6 and September 30, 2024, with documented informed consent acquired from each participant before study commencement.

### Sample size estimation

Sample size calculations were performed using G*Power software (version 3.1), with a significance level (α) set at 0.05 and a statistical power (1-β) of 0.8. The minimum detectable effect size was established as d = 0.5 according to previous research conducted by Yvonne Tran and colleagues [28]. Accounting for an anticipated 10% participant attrition rate, the final calculated minimum total sample size yielded a requirement of 37 study participants.

### Design and program

To minimize circadian rhythm influences, experimental sessions were scheduled consistently from 16:00–18:00 hours Prior to the formal experiment, each participant underwent a standardized 3-minute practice session to ensure task comprehension and procedural familiarity. The experimental protocol initiated with a 3-minute resting-state EEG recording, directly succeeded by baseline fatigue evaluation using the Visual Analog Scale for Fatigue (VAS-F). Then, participants engaged in a 40-minute Stroop task, after which they filled out the VAS-F again and recorded 3-minute resting-state EEG.

Ethical approval for the investigation was granted by the Ethics Committee of Beijing Rehabilitation Hospital, Capital Medical University (Ethics Approval Number: 2023bkky-079–001), in compliance with the ethical principles outlined in the Declaration of Helsinki, and registered with the Clinical Trial Registration Center (Registration Number: ChiCTR2400092212).

#### Cognitive fatigue induction paradigm.

The Stroop task represents a validated neuropsychological assessment paradigm that activates multiple higher-order cognitive functions, including response inhibition, attentional control, and information processing speed, all exhibiting marked susceptibility to fatigue-related impairments. Given its established sensitivity in detecting cognitive fatigue manifestations, this paradigm has been extensively employed for experimental fatigue induction [29,30]. Following the methodological approach developed by Bruijel et al. [31], this investigation implemented an adapted 40-minute Stroop task for effective fatigue induction. The specific experimental procedure involved randomly presenting trials with both consistent and inconsistent text colors, during which participants were required to suppress the automatic processing of the semantic meaning of text while accurately identifying the color of the text and providing an appropriate button response.

#### Subjective assessment of cognitive fatigue.

Developed by Lee et al. [32], the VAS-F serves as a validated psychometric instrument for assessing the subjective experience of cognitive fatigue. The scale comprises two complementary dimensions: a fatigue subscale employing a 10-cm VAS ranging from 0 (indicating no fatigue) to 10 (representing extreme fatigue), along with an energy subscale also employing a 10-cm VAS ranging from 0 (indicating no energy) to 10 (representing being full of energy). Participants complete the self-assessment by indicating their current state along each continuum. Fatigue states are characterized by elevated fatigue subscale scores and corresponding reduced energy subscale scores. The VAS-F has gained widespread adoption for self-assessing short-term fatigue owing to its demonstrated psychometric reliability, straightforward administration protocol, and efficient completion time. Moreover, this assessment instrument finds extensive application across clinical research fields such as neurological rehabilitation assessment, chronic disease management, and the monitoring of psychiatric symptoms [33,34].

### EEG data acquisition and preprocessing

**Fig 1:**
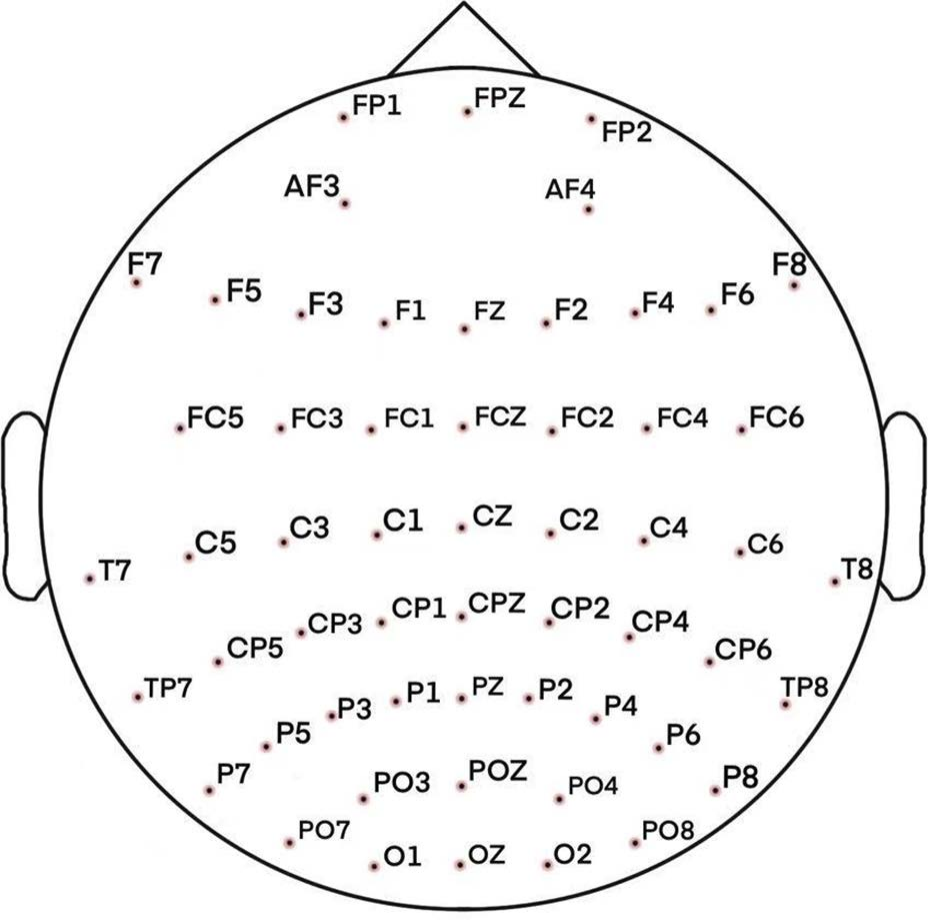
Distribution of EEG electrode positions.

EEG recordings were collected under a controlled a quiet environment, while participants remained alert yet, relaxed state with eyes open. A 64-channel Neuroscan system, arranged following the standardized international 10–20 electrode configuration, captured continuous EEG signals at 500 Hz sampling frequency. Electrode impedance was maintained below 10 kΩ to ensure signal quality. Data preprocessing was conducted using MATLAB (2020B) and the EEGLAB toolbox (2024), which involved epoch rejection, re-referencing, filtering, interpolation of bad channels, and Independent Component Analysis (ICA) for artifact removal. The final dataset comprised 56 channel signals, as depicted in Fig 1.

### Statistical analysis

#### VAS-F.

Statistical analysis of the VAS-F derived subjective fatigue measures was conducted utilizing SPSS Statistics software (version 23.0). Normality assumptions were verified using the Shapiro-Wilk test. Comparative analysis of pre-task and post-task VAS-F subscale scores was performed using paired-sample t-tests for normally distributed data) and Wilcoxon signed-rank tests for non-normally distributed data, establishing fatigue-related changes, with statistical significance set at *p* < 0.05.

#### PSD.

The Welch method was implemented for spectral power analysis of preprocessed EEG data. This approach employs a segmentation strategy with 50% overlapping Hanning windows to enhance spectral estimation reliability. For each windowed segment, PSD estimates were computed through Fast Fourier Transform (FFT), followed by ensemble averaging across segments to minimize spectral variance and reduce noise contamination. Following spectral decomposition, band-specific power was extracted for standard frequency bands: θ (4–8 Hz), α (8–13 Hz), and β (13–30 Hz). Whole-brain mean PSD were then derived through channel-wise averaging of spectral power for each designated frequency band.

Statistical analyses were performed using SPSS Statistics software (version 23.0). Normality assumptions were verified using the Kolmogorov-Smirnov test.

For normally distributed data, parametric within-subject analyses employed paired-sample t-tests with significance set at *p* < 0.05 and effect magnitude quantified via *Cohen’s d*:


$$d=M_{diff}/S_{diff}$$

*M*_*diff*_ denotes the mean difference between paired observations, and *S*_*diff*_ represents the standard deviation of the paired differences.

For non-normally distributed data, non-parametric statistical analyses were conducted using the Wilcoxon signed-rank test with significance set at *p* < 0.05 and effect size (*r*) determined through the following equation:


$$r=Z/\sqrt{}N$$

*Z* represents the standardized test statistic derived from the Wilcoxon signed-rank test, and *N* denotes the total number of paired observations.

The conversion between *Cohen’s d* and *r* effect size metrics was established through the following relationship:


$$r=d/\sqrt{}d^{2}+4$$

The frequency band exhibiting the maximum effect size magnitude emerged as the optimal biomarker for cognitive fatigue detection, exhibiting superior sensitivity compared to other bands.

To control for the risk of false positives due to multiple comparisons, Bonferroni correction was implemented for significance levels when analyzing PSD variations within the α, β and θ frequency bands before and after cognitive fatigue. The specific methodology is as follows: (1) Setting the adjusted threshold: *α’ = α/m,* where *α’* represents the adjusted threshold, *α* is the original significance level (*α = 0.05*), and *m* is the number of tests (*m* *=* *3*), resulting in a final adjusted threshold of *α’* = 0.0167.

(2) Statistical significance was determined using the following criterion: PSD change within a given frequency band achieved statistical significance when the uncorrected p-value for the observed changes fell below the threshold of 0.0167.

#### Construction of the wPLI brain network matrix.

The phase information was derived from frequency-domain transformed signals using Hilbert Transform. Phase synchronization between each pair of EEG channels was assessed using the wPLI, with the specific computation for channels *i* and *j* performed as follows:


wPLI(i,j)=|Σ[sin(Δφ(i,j,k))]/N|

*i* and *j* represent distinct EEG channels, *k* indexes temporal samples, and *N* denotes the total number of time points.

Following the wPLI computation for all unique channel pairs, a symmetric 56 × 56 association matrix was constructed, with each element (*i,j*) containing the *wPLI* value between channels *i* and *j*.

#### Graph theory analysis.

The functional connectivity matrix was subjected to proportional thresholding to retain the strongest functional connections while preserving network sparsity characteristics. Graph-theoretical analysis was employed to characterize network topology, evaluating both global and local network properties. At the global level, the following metrics were examined: global efficiency (Eg), local efficiency (Eloc), clustering coefficient (Cp), shortest path length (Lp), and small-world property (Sigma). Local network metrics comprised nodal degree centrality (DC) and nodal efficiency (NE).

Statistical analyses were conducted using SPSS Statistics software (version 23.0). The normality assessment of global and local graph theory metrics was performed using the Shapiro-Wilk test for the following regions: frontal, fronto-central, central, centro-parietal, parietal, and parieto-occipital. However, the normality evaluation for local graph theory metrics in prefrontal, occipital, temporal, and temporoparietal regions was omitted due to insufficient sample size (N ≤ 3). Within-subject comparisons of pre-fatigue and post-fatigue network metrics were performed using paired-sample t-tests for normally distributed data or Wilcoxon signed-rank tests for data that are non-normally distributed or for which normality tests have not been conducted, with significance set at p < 0.05.

The statistical significance of alterations in both global and local graph theory metrics before and after cognitive fatigue was evaluated through application of Bonferroni correction. The adjusted significance thresholds were established at *α’* = 0.01 for global metrics and *α’* = 0.005 for local metrics. The statistical significance was determined by comparing each original *p*-value against these adjusted thresholds, with metric changes deemed significant exclusively when *p*-values fell below the respective *α’* values.

## Results

### Demographics

A cohort of 48 participants was enrolled in this study, with 31 female (64.6%) and 17 male (35.4%) subjects. Complete demographic data are presented in Table 1.

**Table 1:** Demographic characteristics (N = 48).

Characteristics	Mean (SD) or N (%)
Age (years)	24.17 (2.68)
Sex	
Male	17 (35.4%)
Female	31 (64.6%)
Education (years)	16.63 (2.21)
Sleep duration (hours)	7.52 (0.42)

### Subjective fatigue assessment results

Shapiro-Wilk normality testing revealed non-normal distribution characteristics (*p *< 0.05) for VAS-F fatigue component score variations (*p* = 0.004) following cognitive fatigue intervention. In contrast, the changes in scores for the energy component of the scale (*p* = 0.785) demonstrated normal distribution properties (*p* > 0.05).

**Fig 2:**
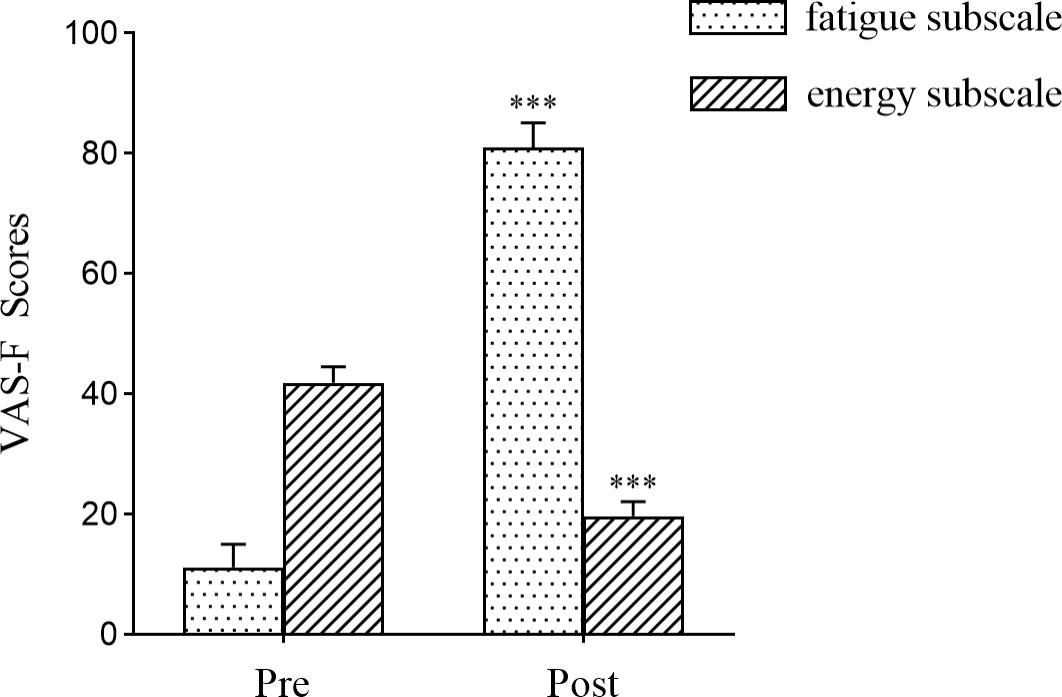
Task-induced alterations in VAS-F.

Quantitative analysis of VAS-F measurements revealed significant task-induced alterations in subjective fatigue states. Fig 2, displayed a pronounced elevation in the fatigue subscale scores post-Stroop task compared to baseline measurements (pre-task: 11.167 ± 3.821, post-task: 80.917 ± 4.187; *p* < 0.001), indicating statistically significant variation. Conversely, the energy subscale scores exhibited a significant reduction after the task (pre-task: 41.833 ± 2.664, post-task: 19.688 ± 2.408; *p* < 0.001). These bidirectional variations in subjective fatigue assessments confirmed the successful induction of cognitive fatigue through the 40-minute Stroop task.

### Spectral power analysis results

The results of the Kolmogorov-Smirnov normality testing demonstrated normal distribution characteristics (*p* > 0.05) for the changes in the PSD in both the α band (*p* = 0.200) and the θ band (*p* = 0.200) before and after the cognitive fatigue intervention. In contrast, the PSD changes in the β band (*p* = 0.016) exhibited non-normal distribution properties (*p* < 0.05).

**Fig 3:**
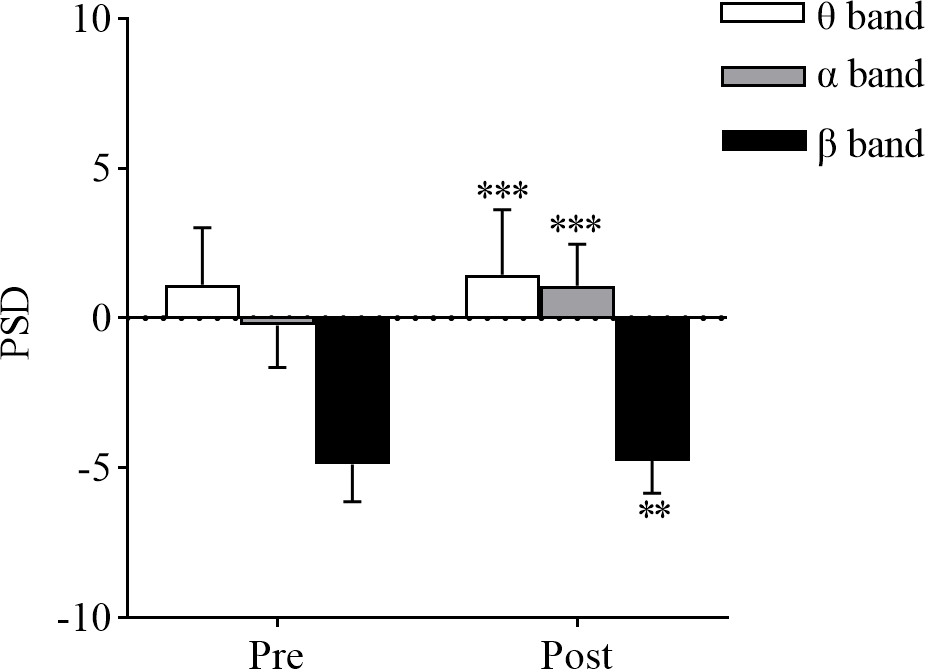
Fatigue-induced alterations in PSD.

Quantitative comparisons of fatigue-induced alterations in neural oscillations was conducted through paired-sample t-tests of PSD within the α and θ frequency bands. As illustrated in Fig 3, the results revealed a statistically significant elevation in α-band power following cognitive fatigue (pre-fatigue: −0.241 ± 1.398, post-fatigue: 1.097 ± 1.370; *p* < 0.001, Cohen’s d = 4.23, 95% CI [−1.42, −1.25], r = 0.90). θ-band power demonstrated significant elevation (pre-fatigue: 1.126 ± 1.875, post-fatigue: 1.461 ± 2.142; p < 0.001, Cohen’s d = 0.81, 95% CI [0.23, 0.45], r = 0.38). For the β band, non-parametric analysis using the Wilcoxon signed-rank test showed significant power elevation following cognitive fatigue (pre-fatigue: −4.881 ± 1.228, post-fatigue: −4.757 ± 1.068; p = 0.004, r = − 0.27). Statistical analysis revealed that the original p-values for the changes in PSD across the three frequency bands remained below the corrected significance level (α’ = 0.0167), demonstrating statistically meaningful alterations The effect sizes for both the α and θ frequency bands exceeded the conventional threshold for moderate effects (|r| > 0.3) [35], indicating that the oscillatory activity changes in these bands are statistically significant. In contrast, the effect size for the β frequency band fell below the conventional threshold, implying potentially nonsignificant spectral modifications in this frequency range.

### Network topology analysis of α-band wPLI connectivity

Comparative effect size analysis of PSD alterations among the θ, α, and β frequency bands demonstrated maximal impact within the α band (Cohen’s d = 1.10, r = 0.48), indicating a superior sensitivity of α oscillations to cognitive fatigue induced by the Stroop task. This observation prompted the development of a wPLI functional connectivity network specifically in the α frequency range. Subsequent application of graph-theoretical analysis enabled systematic examination of fatigue-induced modifications in both global and local network topology.

#### Global network topology metrics.

The results of the Shapiro-Wilk normality test indicated that the global graph theory metrics (Eloc: *p* = 0.135; Cp: *p* = 0.957) demonstrated normality (*p* > 0.05), whereas Eg (*p* = 0.003), Lp (*p* = 0.001), and Sigma (*p* = 0.006) did not meet the criteria for normality (*p* < 0.05).

**Fig 4:**
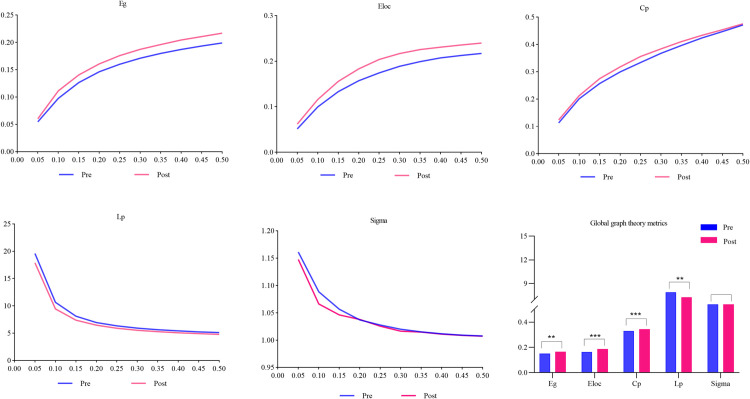
Fatigue-induced alterations in global network topology metrics.

The threshold-dependent analysis, as depicted in Fig 4, demonstrated consistent relationships between connectivity thresholds network metrics: Eg, Eloc, and Cp displayed positive correlations with increasing threshold values, while Lp and sigma exhibited inverse relationships, with these patterns remaining unaffected by fatigue state.

Network topology comparison between pre-fatigue and post-fatigue induction showed significant reorganization of functional connectivity. There were significant increases in Eg (pre-fatigue: 0.152 ± 0.047; post-fatigue: 0.166 ± 0.050; *p* = 0.005), Eloc (pre-fatigue: 0.164 ± 0.055; post-fatigue: 0.187 ± 0.059; *p* < 0.001), and Cp (pre-fatigue: 0.331 ± 0.115; post-fatigue: 0.344 ± 0.113; *p* < 0.001) following cognitive fatigue. In contrast, the Lp (pre-fatigue: 7.897 ± 4.444; post-fatigue: 7.270 ± 3.993; *p *= 0.005) and Sigma (pre-fatigue: 1.043 ± 0.049; post-fatigue: 1.038 ± 0.043; *p *= 0.022) markedly decreased. Following multiple comparisons correction, four network metrics (Eg, Lp, Eloc, and Cp) exhibited significant changes before and after cognitive fatigue (*p *<* *α’ = 0.01). However, Sigma did not show statistically significant changes before and after cognitive fatigue (*p* > α’ = 0.01).

#### Local graph theoretical metrics.

The results of the Shapiro-Wilk normality test indicated normal distribution characteristics for local graph theory metrics (DC and NE) across six cortical regions: frontal, fronto-central, central, centro-parietal, parietal, and parieto-occipital (detailed results presented in Tables 2 and 3).

**Table 2:** Changes in NE between pre-fatigue and post-fatigue.

Brain Regions	Pre-fatigue (Mean ± SD)	Post-fatigue (Mean ± SD)	S-W test	*p*
Prefrontal Area F1, FZ, F2	0.064 ± 0.001	0.069 ± 0.001	—	*p* > 0.05
Frontal Area F7, F5, F3, F1, FZ, F2, F4, F6, F8	0.069 ± 0.002	0.076 ± 0.001	*p* = 0.831	*p* < 0.001^***^
Fronto-central Area FC5, FC3, FC1, FCZ, FC2, FC4, FC6	0.072 ± 0.002	0.079 ± 0.001	*p* = 0.965	*p* < 0.001^***^
Central Area C5, C3, C1, CZ, C2, C4, C6	0.072 ± 0.003	0.081 ± 0.002	*p* = 0.440	*p* < 0.001^***^
Centro-parietal Area CP5, CP3, CP1, CPZ, CP2, CP4, CP6	0.073 ± 0.003	0.081 ± 0.002	*p* = 0.137	*p* < 0.001^***^
Parietal Area P7, P5, P3, P1, PZ, P2, P4, P6, P8	0.071 ± 0.004	0.075 ± 0.006	*p* = 0.785	*p* = 0.012
Parieto-occipital Area PO7, PO3, POZ, PO4, PO8	0.068 ± 0.004	0.073 ± 0.006	*p* = 0.765	*p* = 0.033
Occipital Area O1, OZ, O2	0.064 ± 0.003	0.069 ± 0.005	—	*p* > 0.05
Temporal Area T7, T8	0.062 ± 0.001	0.075 ± 0.001	—	*p* > 0.05
Tempor-oparietal Area TP7, TP8	0.063 ± 0.002	0.071 ± 0.007	—	*p* > 0.05

**Table 3:** Changes in DC between pre-fatigue and post-fatigue.

Brain Regions	Pre-fatigue (Mean ± SD)	Post-fatigue (Mean ± SD)	S-W test	*p*
Prefrontal Area F1, FZ, F2	1.653 ± 0.019	1.628 ± 0.074	—	*p* > 0.05
Frontal Area F7, F5, F3, F1, FZ, F2, F4, F6, F8	1.800 ± 0.144	1.846 ± 0.017	*p* = 0.364	*p* > 0.05
Fronto-central Area FC5, FC3, FC1, FCZ, FC2, FC4, FC6	1.912 ± 0.170	2.032 ± 0.068	*p* = 0.478	*p* = 0.05
Central Area C5, C3, C1, CZ, C2, C4, C6	1.945 ± 0.216	2.236 ± 0.191	*p* = 0.939	*p* = 0.005
Centro-parietal Area CP5, CP3, CP1, CPZ, CP2, CP4, CP6	2.025 ± 0.223	2.289 ± 0.220	*p* = 0.428	*p* = 0.005
Parietal Area P7, P5, P3, P1, PZ, P2, P4, P6, P8	2.013 ± 0.283	2.162 ± 0.373	*p* = 0.297	*p *> 0.05
Parieto-occipital Area PO7, PO3, POZ, PO4, PO8	1.842 ± 0.235	2.139 ± 0.374	*p* = 0.963	*p* = 0.036
Occipital Area O1, OZ, O2	1.710 ± 0.225	1.949 ± 0.295	—	*p* > 0.05
Temporal Area T7, T8	1.149 ± 0.046	1.977 ± 0.180	—	*p* > 0.05
Tempor-oparietal Area TP7, TP8	1.533 ± 0.085	1.855 ± 0.398	—	*p* > 0.05

**Fig 5:**
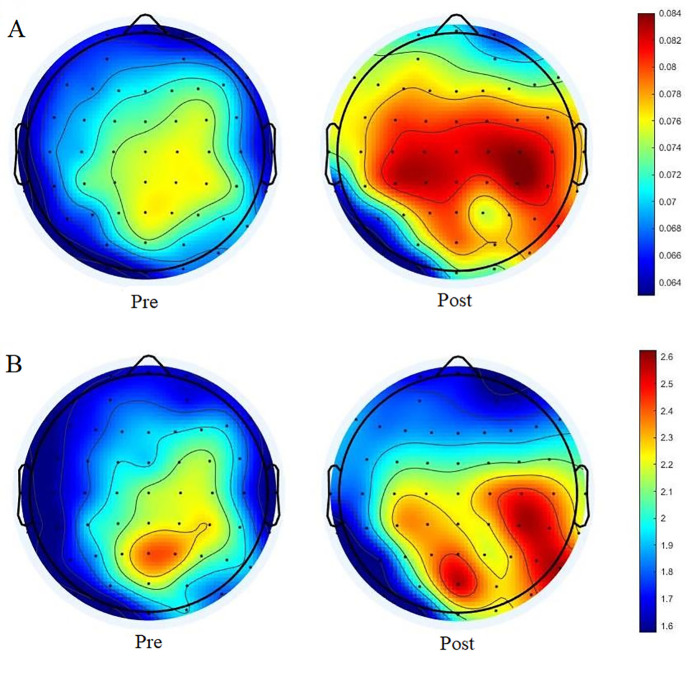
Alterations in local graph-theoretical metrics following cognitive fatigue. A. NE-related brain activation topographies before and after cognitive fatigue. B. DC brain network topology changes before and after cognitive fatigue.

As shown in Fig 5A, NE was systematically quantified across cortical regions to assess the effects of cognitive fatigue on information processing efficiency within brain networks. Comparative analysis demonstrated differential NE between pre-fatigue and post-fatigue conditions (Table 2). Following Bonferroni correction, statistically significant NE variations were restricted to four specific regions: frontal, fronto-central, central, and centro-parietal cortices (all *p* < α’ = 0.005).

DC measurements were systematically computed across all brain regions to characterize alterations in local network connectivity associated with cognitive fatigue. (Fig 5B). Comparative analysis revealed DC variations subsequent to fatigue induction (Table 3). Following Bonferroni correction, there were no significant differences in DC across the brain regions (all *p* ≥ α’ = 0.005).

#### Analysis of correlation.

To explore potential correlations between graph theory metrics and subjective fatigue levels, Spearman correlation analysis was conducted between graph theory variations (including Eg, Eloc, Lp, Cp, and NE of the frontal region, parietal region, central region, and centro-parietal region) and the total score of VAS-F. Analysis outcomes demonstrated statistically significant correlations among specific global graph theory metrics (Eg, Lp,and Cp) and self-reported fatigue severity (Table 4). Specifically, Eg and Lp displayed a positive correlation with subjective fatigue, while Cp showed a negative correlation.

**Table 4:** Correlation analysis between graph theory metrics and VAS-F.

Graph Theory Metrics	*p*	r	Direction of Correlation
Eg	0.005	0.805	Positive Correlation
Lp	0.009	0.774	Positive Correlation
Cp	0041	−0.652	Negative Correlation

## Discussion

The neural mechanisms of cognitive fatigue induced by prolonged cognitive task, performance were examined through EEG-derived functional connectivity features. The findings revealed that: (1) Following cognitive fatigue, the global average PSD exhibited a significant elevation in the α, θ, and β frequency bands, with maximal effect size noted for changes in the α band; (2) wPLI network analysis in the α band indicated fatigue-induced augmentation of Eg, Eloc, and Cp, concomitant with significant Lp attenuation, while Sigma did not show significant statistical differences. Region-specific NE enhancement was detected in frontal, fronto-central, central, and centro-parietal regions, contrasting with the absence of significant DC variations across the brain regions. These experimental results provide quantitative evidence of functional connectivity alterations associated with cognitive fatigue, constructing a theoretical framework for more precise identification of cognitive fatigue states and contributing novel perspectives regarding its neural underpinnings.

### Induction of cognitive fatigue

A critical component of this study was the successful induction of cognitive fatigue. Administration of the 40-minute Stroop test elicited statistically significant elevation in VAS-F fatigue subscale scores accompanied by concurrent reduction in energy subscale measurements. These behavioral outcomes indicate considerable cognitive load and mental exertion during the task. The data confirm the 40-minute Stroop task as a robust method for inducing cognitive fatigue, thereby establishing a reliable behavioral foundation for subsequent investigations.

### Changes in PSD

This study revealed significant alterations in the global average PSD following cognitive fatigue, characterized by a pronounced increase in the α,θ, and β frequency bands. The elevated α-band PSD correlates with heightened cortical inhibition, potentially indicating neural mechanisms that attenuate external information processing to mitigate additional cognitive load during fatigue states [36,37]. θ band neural oscillatory activity shows strong associations with introspective cognition, mnemonic processing, and attentional regulation [38,39]. Under cognitive fatigue conditions, compensatory θ band enhancement may occur to preserve diminished task performance capabilities resulting from depleted cognitive reserves. As a critical indicator of active consciousness, focused attention, and higher cognitive functions, the elevated β-band activity likely represents neural compensatory mechanisms that reinforce attentional focus and task execution control by augmenting β activity [29,36]. The present findings demonstrate that neural oscillatory activity in the θ, α, and β bands constitutes valuable neurophysiological indicators for cognitive fatigue evaluation. Notably, the α band activity displayed the largest effect magnitude, confirming superior sensitivity for detecting cognitive fatigue induced by the Stroop task. Conversely, the β band measurements failed to attain the moderate effect threshold, implying restricted utility in cognitive fatigue assessment paradigms.

The observed alterations in α band neural oscillatory activity following cognitive fatigue induction provide empirical support for the α suppression hypothesis. According to this theoretical framework, low α band power corresponds to engaged neuronal processing, while high α band power indicates the active suppression of neural activity in task-irrelevant brain regions [40,41]. Within the implemented Stroop task paradigm, successful task performance necessitated suppression of automatic word reading while maintaining accurate color discrimination. This inhibitory requirement mediated significant augmentation in α band power, potentially reflecting the prefrontal-mediated top-down control mechanisms implemented through enhanced α oscillatory activity to prevent interference from automatically processed semantic information [39,42]. Previous studies have observed a reduction in α band neural oscillatory activity during cognitive fatigue induced by tasks such as the n-back working memory task and fatigued driving tasks [43,44]. This observed phenomenon potentially stems from constrained inhibitory processing requirements intrinsic to these experimental paradigms, indicative of compromised cognitive resource allocation efficiency and insufficient neural functional compensation. While extant evidence predominantly corroborates α band activity elevation post cognitive fatigue, comprehensive elucidation of the precise regulatory dynamics underlying α band functional activity changes and their neurophysiological implications within fatigue contexts necessitates validation via multimodal experimental approaches.

### Changes in global graph theory metrics

This study further demonstrated that cognitive fatigue induced significant alterations in the topological properties of the brain network, including a marked increase in Eg, Eloc, and Cp, alongside a notable suppression in Lp.

Cp serves as a quantitative metric for evaluating local connectivity density in brain functional networks. Elevated Cp denote intensified regional connectivity and augmented functional modularity among neuronal populations. This parameter exhibits an inverse relationship with neurodegenerative diseases, where diminished Cp consistently manifest as impaired functional integration across cortical regions. Regarding Eloc, this metric provides crucial insights into assessing the local efficiency of information transfer within brain networks. Higher Eloc correlate with superior regional computational efficiency, reflecting both heightened cognitive flexibility and optimized rapid local adaptability. In contrast, decreased Eloc demonstrate compromised local network stability that may precipitate region-specific functional deficits, as documented in prior neuroimaging investigationss [45].The elevated Cp and Eloc in this study indicated a shift toward greater reliance on localized information processing rather than global network integration under fatigued conditions.

Eg is used to quantify the global efficiency of information transfer within the brain network, reflecting the ease of information exchange between different brain regions. Elevated Eg indicate efficient information integration across the network, supporting rapid interregional coordination and processing. In contrast, lower Eg imply compromised long-range connectivity or impaired integrative functionality, potentially contributing to slowed neural signal propagation [46].The observed Eg augmentation subsequent to cognitive fatigue induction may represent neuroadaptive compensation mechanisms, wherein enhanced global information transfer efficiency counterbalances depleted cognitive resources.

Lp constitutes a fundamental parameter brain network topology analysis, serving to measure the efficiency of information propagation pathways. Lower Lp indicates that the brain network possesses efficient direct information transmission capabilities, facilitating rapid information integration. Conversely, higher Lp reflects a reduction in network efficiency [47].The decrease in Lp indicates a tendency toward more direct and streamlined information pathways during fatigue, potentially aimed at conserving energy and facilitating rapid responses to task demands. Such topological modifications likely constitute compensatory neural adaptations to preserve cognitive functionality under conditions of fatigue.

As a principal parameter for evaluating topological optimization in brain functional networks [48], Sigma exhibits no statistically significant variation following cognitive fatigue induction, with results remaining robust after Bonferroni adjustment. This finding suggests that despite the presence of cognitive fatigue, the brain functional network maintains typical small-world topological characteristics, and the balance between local information processing efficiency and global information integration capabilities has not undergone substantial change. The neural architecture retains normative organizational parameters throughout fatigue conditions, showing no deviation toward the pathological configurations characteristic of neuropsychiatric populations.

### Changes in local graph theory metrics

This study analyzed the characteristics of brain functional networks using NE and DC. The NE parameter measures regional information transfer efficiency within neural networks, while higher values denote superior performance as information integrative neural hubs [49,50]. DC evaluates the structural significance of nodes within local networks, where greater DC generally identify principal elements within functional modules [50,51]. The results revealed marked NE enhancement across frontal, fronto-central, central, and centro-parietal regions, suggesting an improvement in the functional efficiency of these areas in information processing and transmission. By contrast, the whole-brain DC metric demonstrated no significant differences, implying that neural compensatory mechanisms predominantly involve refinement of local information processing efficiency rather than macrostructural reorganization, while maintaining the overall stability of fundamental network connectivity. This finding provides new evidence for understanding the mechanisms of brain network reorganization under cognitive states.

The analysis of NE in this study revealed a significant increase in the NE of the frontal, fronto-central, central, and centro-parietal regions. From a functional anatomical perspective, the enhancement of NE in these regions holds significant cognitive ramifications. As the principal cortical substrate for higher-order cognition [52,53], the frontal lobe demonstrates elevated NE values that reflect enhanced information processing efficacy and cross-modal integration capacity, potentially supporting superior information coordination during the execution of complex cognitive tasks. Similarly, the central region exhibits NE increases indicative of refined neural circuitry dedicated to motor control and sensory information processing [54]. The observed NE elevation in the centro-parietal region potentially signifies enhanced spatial cognitive processing and attentional resource distribution [55,56]. These findings support the hypothesis that brain networks reorganize through compensatory restructuring of key nodes to maintain cognitive function during fatigued states [45,57]. Notably, such compensatory network reorganization, which optimizes regional processing efficiency, appears to compromise global integrative functionality, which may subsequently impair performance in cognitively demanding tasks.

The prefrontal cortex, while fundamental for cognitive functions, demonstrates no significant NE changes before and after cognitive fatigue. This observation potentially stems from multiple neurophysiological factors: the α-band frequency neural oscillations primarily reflect cortical inhibition, and the characteristically low intensity of α activity in the prefrontal cortex may limit the sensitivity of α-derived network metrics to detect functional modifications within this region [28].Functioning as the principal neural substrate for advanced cognitive regulation, the prefrontal cortex may maintain the stability of local network functions during initial fatigue phases via adaptive strategies including neural resource redistribution..These hypotheses require empirical verification employing multimodal approaches such as multi-frequency brain network analysis and high-resolution neuroimaging techniques.

### Correlation analysis

The present investigation revealed statistically significant associations between certain graph theory metrics derived from α-band functional brain networks and subjective levels of fatigue, thereby corroborating the diagnostic utility of α-band oscillations in detecting cognitive fatigue. While certain topological measures failed to demonstrate significant correlations, this observation does not preclude the existence of potential underlying relationships with cognitive fatigue manifestations. Several potential explanations may account for these findings: first, dimensional disparities exist between assessment modalities (the VAS-F scale primarily evaluates subjective experiences at the conscious level, while specific neural oscillation metrics from specific brain regions may reflect fatigue accumulation at the unconscious level); second, inter-individual variability could attenuate population-level pattern detection. Subsequent investigations should implement multimodal integration approaches combining behavioral, physiological, and neuroimaging datasets, or alternatively utilize sophisticated computational techniques including machine learning algorithms to substantiate and refine the current findings.

This study indicates that individuals experiencing cognitive fatigue activate neural compensatory mechanisms to sustain performance in cognitive tasks. Existing empirical evidence demonstrates that compensatory neural activation markedly augments cerebral metabolic demands, with documented elevations in both glucose utilization rates and heightened ATP consumption [58,59]. Progressive fatigue development correlates with diminishing compensatory capacity, subsequently manifesting as cognitive deficits characterized by working memory efficiency deterioration, attentional maintenance impairment, and reduced information processing velocity.. This decline may increase the risk of mental health disorders, including anxiety and depression.

The current investigation yields clinically significant implications for cognitive rehabilitation training, necessitating optimization of critical intervention parameters including training duration and intensity thresholds. Implementation of a multimodal evaluation framework integrating subjective fatigue assessments (such as the VAS-F) with objective indicators (such as EEG) is proposed to facilitate real-time protocol modifications. Notably, neurophysiological markers demonstrating enhanced NE in the frontal and parietal regions represent potential novel intervention targets. It is recommended to use fMRI-guided transcranial magnetic stimulation (TMS) in conjunction with cognitive training for targeted interventions. This precision intervention harnesses the effects of neural plasticity while mitigating potential adverse effects of excessive training exposure.

### Limitations and future research directions

Several methodological constraints should be acknowledged in the present investigation. Firstly, a control condition (such as a comparison group not engaged in cognitive tasks under similar experimental conditions) was not included, preventing a complete exclusion of environmental factors affecting the results. Secondly, the experimental protocol omitted quantification of behavioral indices including response latency and task precision measures. While compensatory neural processes may mitigate behavioral performance deterioration and participant learning adaptation capacities could potentially mask fatigue-related effects, the integration of behavioral metrics in subsequent investigations remains imperative for thorough assessment of cognitive fatigue consequences. Finally, the exclusive inclusion of neurotypical participants in the current study warrants consideration, given that pathological cognitive impairments typically involve heterogeneous alterations in both brain structure and functional network connectivity. Future investigations should focus on individuals with cognitive impairments to clarify the mechanisms underlying cognitive fatigue, which is crucial for developing personalized treatment and rehabilitation plans.

## Conclusions

The current investigation identifies that modulations in θ, α, and β frequency band activities serve as significant neural markers for identifying cognitive fatigue, with the α band exhibiting heightened sensitivity to sustained cognitive fatigue induced by the Stroop task. The onset of cognitive fatigue triggers distinct modifications in neural functional connectivity, characterized by compensatory reorganization aimed at enhancing both local and global information transfer efficiency, as well as functional connectivity within key brain regions. However, these compensatory mechanisms may result in an overall reduction in network efficiency. The experimental results establish a robust theoretical foundation for investigating the pathophysiology of cognitive fatigue, while simultaneously informing the development of targeted neurotherapeutic strategies through empirically derived mechanistic insights.

## Supporting information

S1 File Minimal data set. (XLSX)
